# 2′-deoxy-2′-[18F] fluoro-D-glucose positron emission tomography, diffusion-weighted magnetic resonance imaging, and choline spectroscopy to predict the activity of cetuximab in tumor xenografts derived from patients with squamous cell carcinoma of the head and neck

**DOI:** 10.18632/oncotarget.25574

**Published:** 2018-06-19

**Authors:** Lionel Mignion, Sandra Schmitz, Nicolas Michoux, Xavier Caignet, Rose-Marie Goebbels, Anne Bol, Marie-Aline C. Neveu, Vincent Grégoire, Thierry Duprez, Renaud Lhommel, Fréderic Amant, Els Hermans, Benedicte F. Jordan, Jean-Pascal Machiels

**Affiliations:** ^1^ Biomedical Magnetic Resonance Research Group, Louvain Drug Research Institute, Université Catholique de Louvain, Brussels, Belgium; ^2^ Institut Roi Albert II, Service d’Oncologie Médicale, Cliniques Universitaires Saint-Luc and Institut de Recherche Clinique et Expérimentale, Université catholique de Louvain, Brussels, Belgium; ^3^ Department of Radiology and Medical Imaging, Cliniques universitaires Saint-Luc, Université Catholique de Louvain, Brussels, Belgium; ^4^ Center for Molecular Imaging, Radiotherapy and Oncology, Institut de Recherche Expérimentale et Clinique, Université Catholique de Louvain, Brussels, Belgium; ^5^ Department of Nuclear Medicine, Cliniques Universitaires Saint-Luc and Institut de Recherche Clinique et Expérimentale (POLE MIRO), Université Catholique de Louvain, Brussels, Belgium; ^6^ Department of Oncology, Gynecologic Oncology, KU Leuven (University of Leuven), Leuven, Belgium

**Keywords:** cetuximab, FDG-PET, DW-MRI, patient-derived tumor xenograft, head and neck cancer

## Abstract

We investigated changes on 2′-deoxy-2′-[18F]fluoro-D-glucose positron emission tomography (^18^FDG-PET), diffusion-weighted magnetic resonance imaging (DW-MRI), and choline spectroscopy as early markers of cetuximab activity in squamous cell carcinoma of the head and neck (SCCHN).

SCCHN patient-derived tumor xenografts models were selected based on their cetuximab sensitivity. Three models were resistant to cetuximab and two were sensitive (one was highly sensitive and the other one was moderately sensitive). Cetuximab was infused on day 0 and 7. Maximal standardized uptake values (SUVmax), apparent diffusion coefficient (ADC), and total choline pool were measured at baseline and at day 8. To investigate the possible clinical relevance of our pre-clinical findings, we also studied the SUVmax and ADC modifications induced by cetuximab in five patients.

Cetuximab induced a significant decrease in SUVmax and an increase in ADC at day 8 compared to baseline in the most cetuximab-sensitive model but not in the other models. At day 8, in one resistant model, SUVmax was decreased compared to baseline and was significantly lower than the controls. Choline spectroscopy was not able to predict cetuximab activity. The five patients treated with cetuximab had a ^18^FDG-PET partial response. One patient had a partial response according to RECISTv1.1. Interestingly, this last had also an increase in ADC value above 25%.

Our preclinical data support the use of PDTX to investigate imaging techniques to detect early treatment response. Our pre-clinical and clinical data suggest that DW-MRI and ^18^FDG-PET should be further investigated to predict cetuximab activity.

## INTRODUCTION

Squamous cell carcinoma of the head and neck (SCCHN) is the seventh most common cancer worldwide with approximately 630,000 new patients diagnosed annually [1]. Around 60% of patients with SCCHN present with locally advanced disease (LA-HNSCC) and require a multimodal treatment approach that includes either definitive chemoradiation (CRT) or surgery followed by radiation therapy (RT) or CRT. Despite this aggressive strategy, more than 50% of these patients develop local and/or regional recurrences, and approximately 20% develop distant metastases [2, 3]. In the metastatic and/or recurrent setting, platinum agents are still the most active cytotoxic compounds, but the benefit is modest with a median overall survival (OS) that does not exceed nine months [3].

The Epidermal Growth Factor Receptor (EGFR) is a transmembrane tyrosine kinase receptor belonging to the HER/erbB family. Up to 90% of SCCHN express high levels of EGFR [4]. The overexpression of EGFR is associated with poor prognosis, radioresistance, and chemoresistance [5–7]. Cetuximab is a chimeric IgG1 monoclonal antibody (mAb) that specifically binds to the EGFR with high affinity. Cetuximab improves OS when associated with radiation therapy in locally advanced SCCHN, or with platinum-based chemotherapy in incurable disease [8, 9]. However, with single agent objective response rates between 6% and 13%, only a minority of patients derives long-term benefit from anti-EGFR mAbs [9, 10]. In contrast to colon cancer, where RAS mutations predict treatment resistance [11], little is known about the potential mechanisms of cetuximab resistance in SCCHN. To date, no predictive biomarkers able to select patients for anti-EGFR therapies have been validated, and little is known about primary and acquired resistance mechanisms.

Early prediction of treatment resistance could hasten the discontinuation of ineffective treatment and thus reduce unnecessary toxicity. The Response Evaluation Criteria in Solid Tumors (RECISTv1.1) is the current standard to assess objective response in the clinic [12]. However, RECISTv1.1 has some limitations. First, several weeks are generally required to classify a patient as a responder (complete response or partial response) or a non-responder (progressive disease), and RECIST cannot be used as an early marker of response or resistance. Second, not all metastatic sites are measurable according to RECIST (i.e. bone or peritoneal metastases). Finally, RECIST is inadequate when it comes to assessing response to novel therapeutics that target a specific metabolic pathway rather than being cytotoxic. Some of these drugs can be clinically active but may not result in tumor size reduction [13]. Molecular imaging techniques such as 2′-deoxy-2′-[18F] fluoro-D-glucose positron emission tomography (^18^FDG-PET), diffusion-weighted magnetic resonance imaging (DW-MRI), or choline spectroscopy have the potential to detect metabolic changes that will occur earlier than measurable changes in tumor size.

An early ^18^FDG-PET response has been associated with anti-EGFR therapy outcome [14–16]. By comparing the tracer uptake before and after treatment, tumor response can be evaluated [17]. DW-MRI has also been used to detect early changes after standard or targeted therapies [13, 18–19]. Cell death in response to therapies can precede size change and increase the mobility of water molecules in the tissue environment. DW-MRI may therefore be an early biomarker of response. Choline 1H-Magnetic Resonance Spectroscopy (1H-MRS) is also showing promise to assess response to different classes of molecular therapies with aberrant choline phospholipid metabolism being described in a wide variety of cancers [20]. For tumor staging and treatment effect monitoring, the addition of spectroscopy to standard MRI methods can significantly increase the specificity and the sensitivity of the method [21]. However, these different functional imaging techniques have not been prospectively validated and have only rarely been investigated in head and neck cancer.

The pre-clinical mouse models used to study innovative imaging tools to predict treatment response also have important limitations. Currently, most studies have been performed with high-passage commercially available cell lines together with xenograft models derived from these cell lines. However, these models only partially recapitulate the genetic features and tumor heterogeneity from patients with cancer. Patient-derived xenografts, where the tumor is derived directly from a patient’s biopsy, are better at maintaining the morphological and molecular markers of the source tumors over time, even after serial passages across several generations of mice. They are therefore better predictive models [22].

In this study, we investigated early changes on 2′-deoxy-2′-[18F]fluoro-D-glucose-PET, DW-MRI, and choline spectroscopy as early markers of cetuximab response or resistance in SCCHN patient-derived tumor xenografts.

## RESULTS

### Sensitive and resistant SCCHN PDTX models

**Figure 1:**
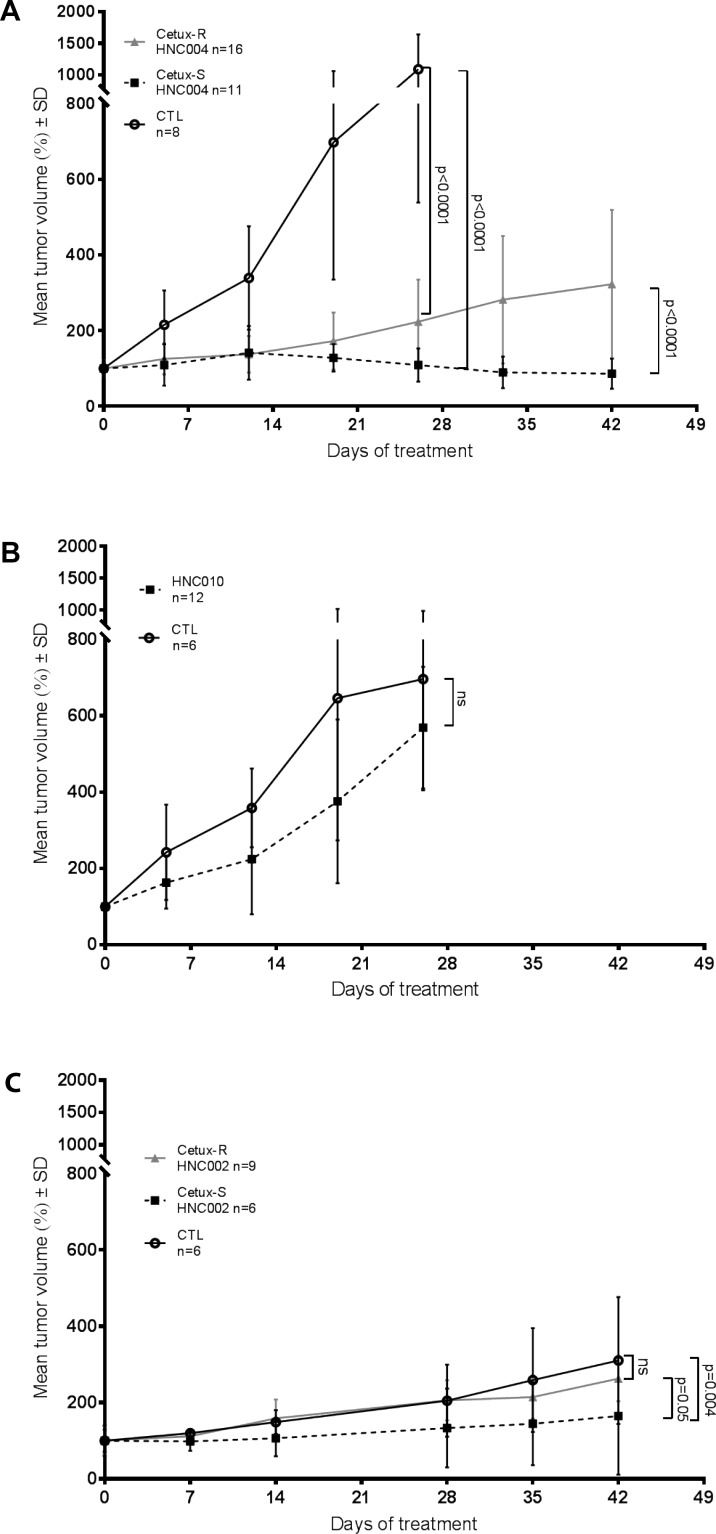
Tumor growth of the different patient-derived tumor xenograft models (**A**) Tumor growth of the HNC004 derived models. CTL = Cetux-S HNC004 control mice treated with saline solution; SD = standard deviation; Cetux-S HNC004 = Cetux-S HNC004 mice treated with cetuximab (30 mg/kg once a week); Cetux-R HNC004 = Cetux-R HNC004 mice treated with cetuximab (30 mg/kg once a week). (**B**) Tumor growth of the HNC010 model. ns = non-significant; CTL = HNC010 control mice treated with saline solution; HNC010 = HNC010 mice treated with cetuximab (30 mg/kg, once a week). (**C**) Tumor growth of the HNC002 derived models. CTL = Cetux-S HNC002 control mice treated with saline solution; Cetux-S HNC002 = Cetux-S HNC002 mice treated with cetuximab (30 mg/kg once a week); Cetux-R HNC002 = Cetux-R HNC002 mice treated with cetuximab (30 mg/kg once a week).

To study if the different imaging techniques could predict early sensitivity or resistance to cetuximab, we chose five SCCHN PDTX models. Two models were sensitive to cetuximab (Cetux-S HNC002 and Cetux-S HNC004), one had primary resistance to cetuximab (HNC010), and two had acquired resistance to cetuximab (Cetux-R HNC002 and Cetux-R HNC004) (Figure 1).

HNC010 was primarily resistant to cetuximab with no difference in tumor growth between controls and cetuximab treated mice. Cetux-S HNC004 was sensitive to cetuximab, and in this model cetuximab decreased the tumor volume over time. In Cetux-S HNC002, cetuximab was not able to decrease tumor volume but had moderate activity, as detected by a significant delay in tumor growth compared with controls.

The Cetux-R HNC002 and Cetux-R HNC004 models both had acquired resistance to cetuximab. These two models were obtained by treating the two sensitive cetuximab models (Cetux-S HNC002 and Cetux-S HNC004) chronically with cetuximab until treatment resistance occurred, as described in the methodology section. In Cetux-S HNC002 and Cetux-S HNC004, cetuximab resistance occurred after two and six months of treatment, respectively (data not shown). In these cetuximab acquired resistant-generated models, tumor grew significantly faster when treated with cetuximab compared to corresponding Cetux-S models, but the speed of growth was still slower than that observed in the Cetux-S models treated with a saline solution (CTL) (Figure 1).

### Imaging results

Imaging techniques were performed at baseline, day 1, and day 8. Cetuximab was injected at day 0 and day 7. The results obtained on day 1 are shown in the (Supplementary Figures 1 and 2) but they were not significant.

### Change in imaging parameters between baseline and day 8 (Figure 2)

**Figure 2:**
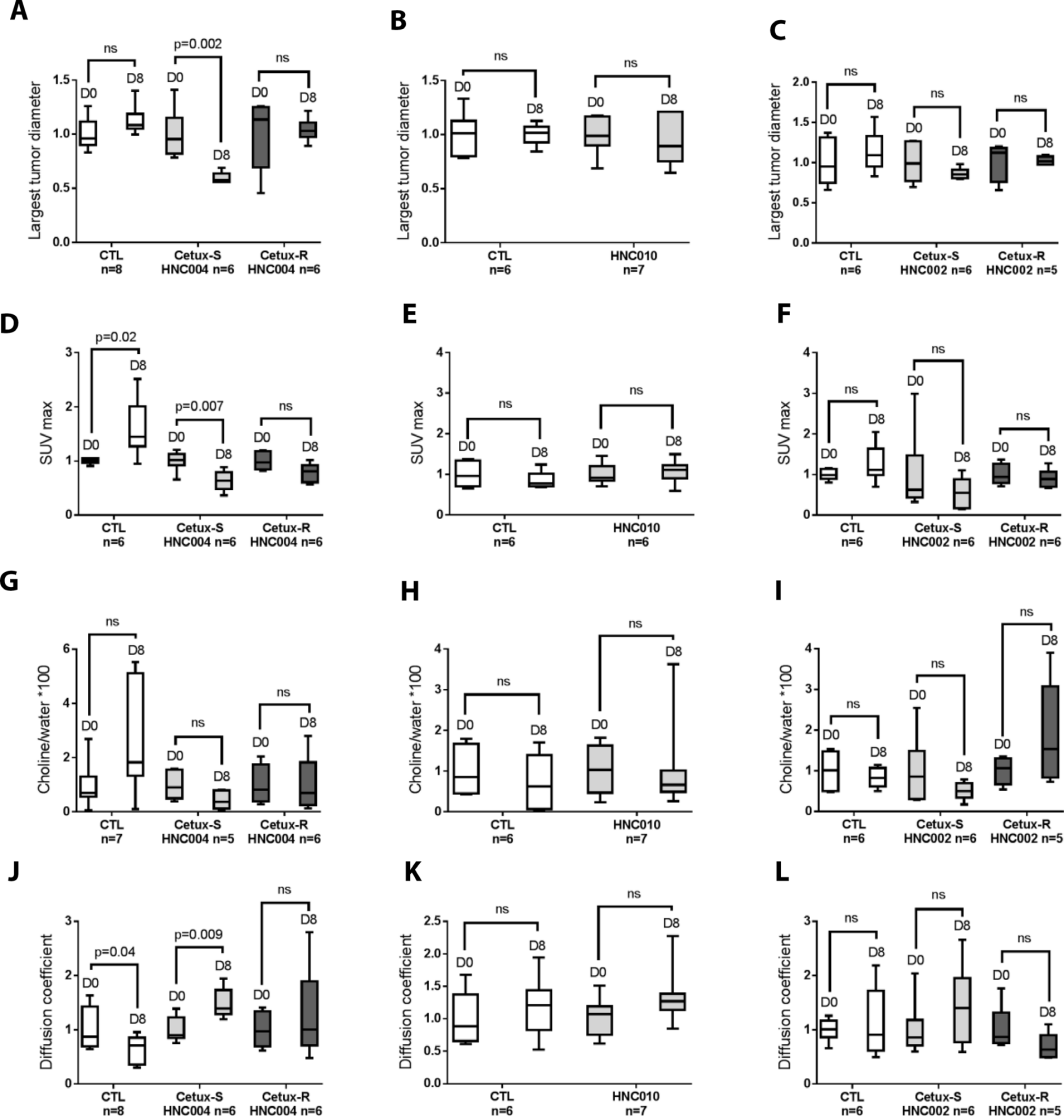
Change in imaging parameters between baseline and day 8 in each model (Box Plot) (**A**–**C**) Modifications of the largest tumor diameter between baseline and day 8 in each group. (**D**–**F**) Evolution of SUV max (standard uptake value) at day 8 compared to baseline in the different models. (**G**–**I**) Changes in Choline/water^*^100 (total choline to water ratio inside the tumor) between day 0 and day 8 in each model. (**J**–**L**) Evolution of apparent diffusion coefficient inside the tumor at day 8 compared to baseline. CTL = control mice of each model treated with saline solution; Cetux-S HNC004 = Cetux-S HNC004 mice treated with cetuximab; Cetux-R HNC004 = Cetux-R HNC004 mice treated with cetuximab; HNC010 = HNC010 mice treated with cetuximab; Cetux-S HNC002 = Cetux-S HNC002 mice treated with cetuximab; Cetux-R HNC002 = Cetux-R HNC002 mice treated with cetuximab. Thirty mg/kg of cetuximab was given intraperitoneally on day 0 and 7.

First, we investigated if cetuximab induced significant modifications between the different imaging parameters between baseline and day 8 in each model.

We only observed statistically significant modifications in the most cetuximab-sensitive model, Cetux-S HNC004, which showed a decrease in the largest tumor diameter (measured on MRI) and SUVmax, as well as an increase in ADC, at day 8 compared to baseline (Figure 2A, 2D and 2J).

No significant modifications of the imaging parameters were found in the three resistant models (HNC010, Cetux-R HNC002, Cetux-RHNC004) and in Cetux-S HNC002 model, the model in which cetuximab had only moderate activity (tumor growth stabilization, Figure 1). Of note, SUVmax increased significantly while ADC decreased significantly in the untreated Cetux-S HN004 model (CTL) (Figure 2D and 2J).

No significant modifications of tCho pool were found in any of the treatment and control groups regardless of the models investigated (Figure 2G–2I).

### Comparison of the imaging parameters between different groups in each model at day 8 (Figure 3)

**Figure 3:**
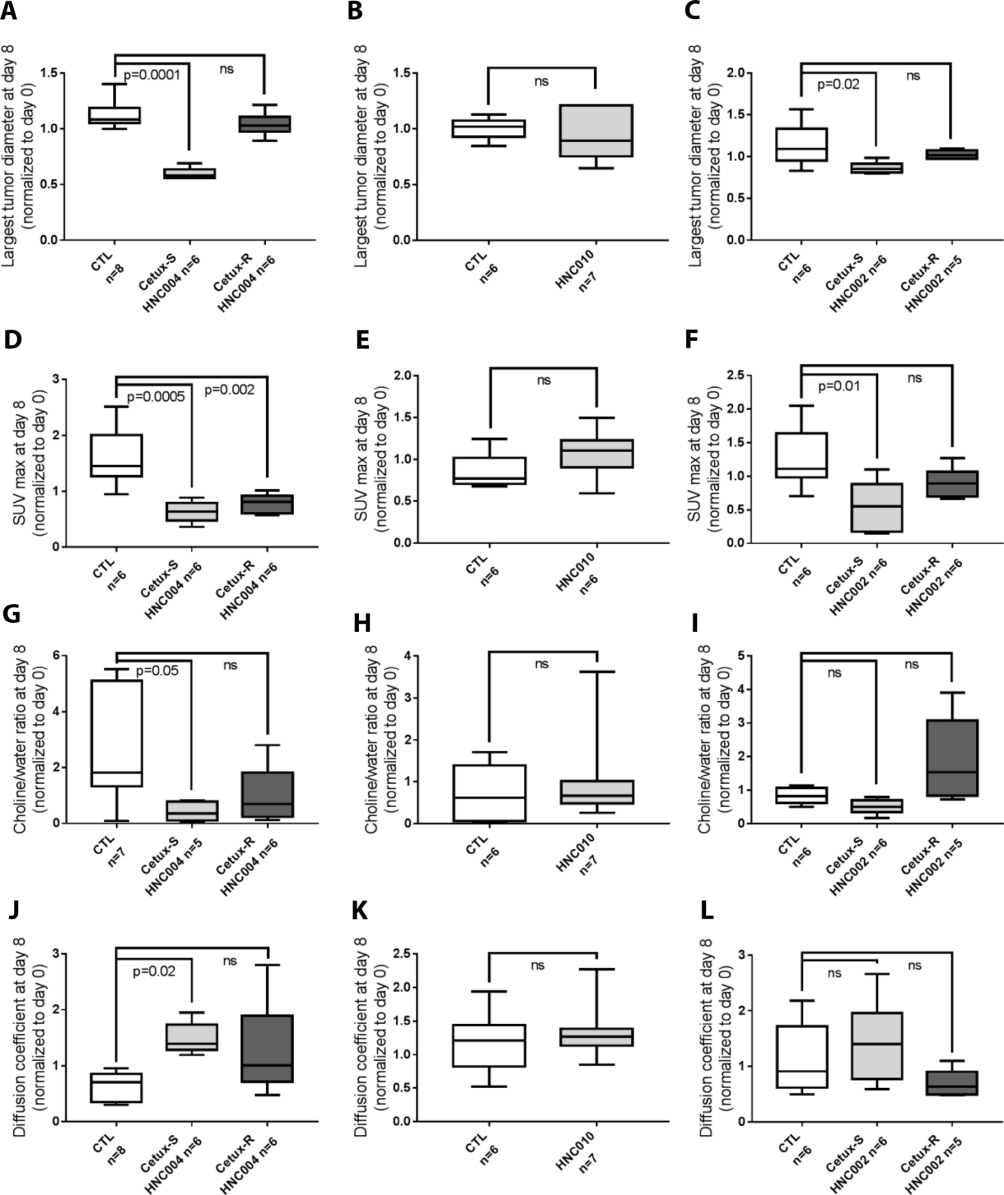
Comparison of the imaging parameters between different groups in each model at day 8 (Box Plot) (**A**–**C**) In each model, comparison of the largest tumor diameter at day 8 between different groups and their respective control. (**D**–**F**) In each model, comparison of the SUV max (standard uptake value) at day 8 between different groups. (**G**–**I**) At day 8, comparison of the choline/water ratio between different groups. (**J**–**L**) Comparison of the apparent diffusion coefficient at day 8 between different groups and their respective control. CTL = control mice of each model treated with saline solution; Cetux-S HNC004 = Cetux-S HNC004 mice treated with cetuximab; Cetux-R HNC004 = Cetux-R HNC004 mice treated with cetuximab; HNC010 = HNC010 mice treated with cetuximab; Cetux-S HNC002 = Cetux-S HNC002 mice treated with cetuximab; Cetux-R HNC002 = Cetux-R HNC002 mice treated with cetuximab. Thirty mg/kg of cetuximab was given intraperitoneally on day 0 and 7.

As expected, at day 8, the largest tumor diameter measured by MRI was significantly smaller in Cetux-S HNC004 and Cetux-S HNC002 groups compared to their respective controls. For the resistant models, no difference in the tumor diameters compared to the control mice could be detected at day 8 (Figure 3A–3C).

In the ^18^FDG-PET experiments, SUVmax was significantly lower in the two cetuximab sensitive models (Cetux-S HNC004, and Cetux-S HNC002) compared to their saline solution treated controls. SUVmax was also significantly lower compared with controls in the Cetux-R HNC004 resistant model. No differences in the two other resistant models (HNC010 and Cetux-R HNC002) were detected (Figure 3D–3F).

In the DW-MRI experiments, ADC was significantly higher compared with controls only in Cetux-S HNC004, the most sensitive model. No differences with the controls were observed in the resistant models (HNC010, Cetux-R HNC004 and Cetux-R HNC002) (Figure 3J–3L).

tCho pool was lower after cetuximab in Cetux-S HNC004 and Cetux-S HNC002 compared to their controls at day 8, but this difference was significant only in Cetux-S HNC004 (Figure 3G–3I).

### pEGFR expression

**Figure 4:**
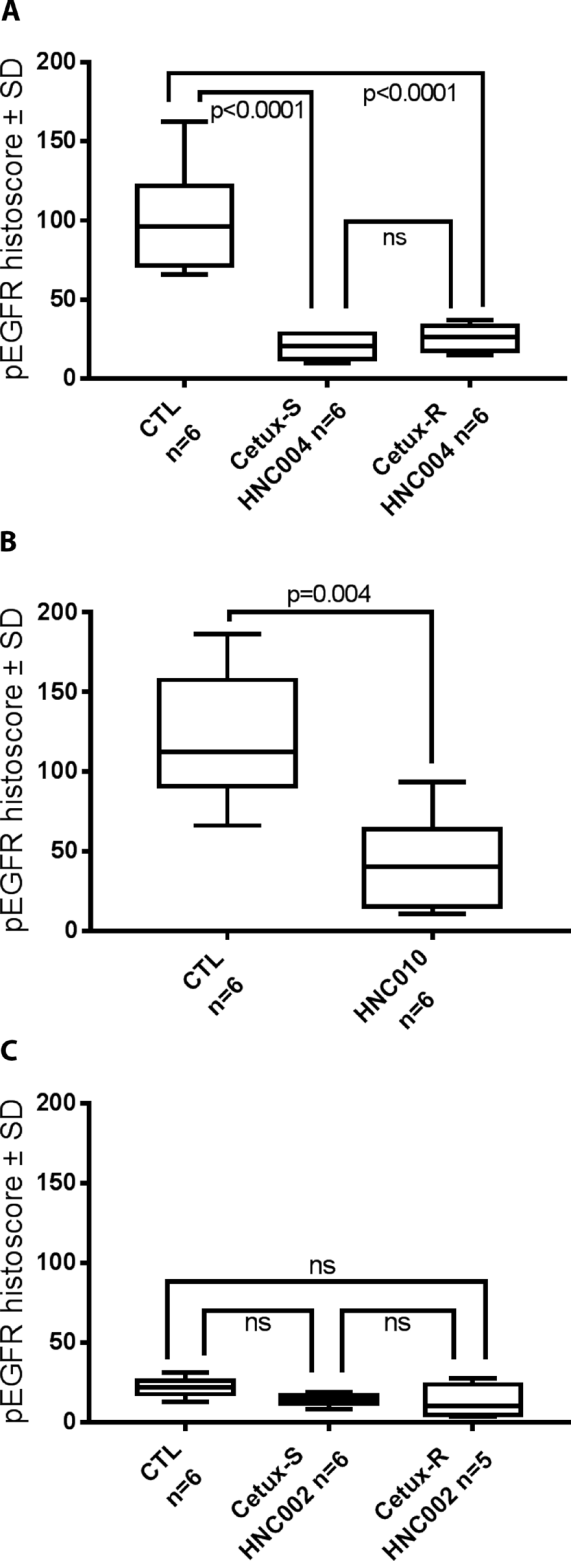
pEGFR histoscore (Box Plot) performed on the tumors harvested at day 8 after sacrificing the mice used in the imaging experiments ns = non-significant. (**A**) HNC004 derived models. CTL = Cetux-S HNC004 control mice treated with saline solution; Cetux-S HNC004 = Cetux-S HNC004 mice treated with cetuximab (30 mg/kg intraperitoneally at day 0 and 7); Cetux-R HNC004 Cetux-R HNC004 mice treated with cetuximab (30 mg/kg intraperitoneally at day 0 and 7). (**B**) HNC010 model. CTL = HNC010 control mice treated with saline solution; Cetux-R = HNC010 mice treated with cetuximab (30 mg/kg intraperitoneally at day 0 and 7). (**C**) HNC002 derived models. CTL = Cetux-S HNC002 control mice treated with saline solution. Cetux-S HNC002 = Cetux-S HNC002 mice treated with cetuximab (30 mg/kg intraperitoneally at day 0 and 7); Cetux-R HNC002 = Cetux-R mice treated with cetuximab (30 mg/kg intraperitoneally at day 0 and 7).

For each model, we compared the expression of pEGFR on the tumors harvested from the sacrificed mice after the imaging assessments performed at day 8. We observed a significant lower expression of pEGFR in all the cetuximab-treated HNC004 and HNC010 models compared to controls, even in the cetuximab-resistant models (Cetux-R HNC004 and HNC010) where the tumors were growing (Figure 4). Interestingly, in all groups (including the controls) derived from the HNC002 models, the level of pEGFR expression was low, and no significant differences were measured between the CTL, Cetux-R and Cetux-S HNC002 groups.

### RECISTv1.1, ^18^FDG-PET, and DW-MRI in five SCCHN patients treated with cetuximab

**Figure 5:**
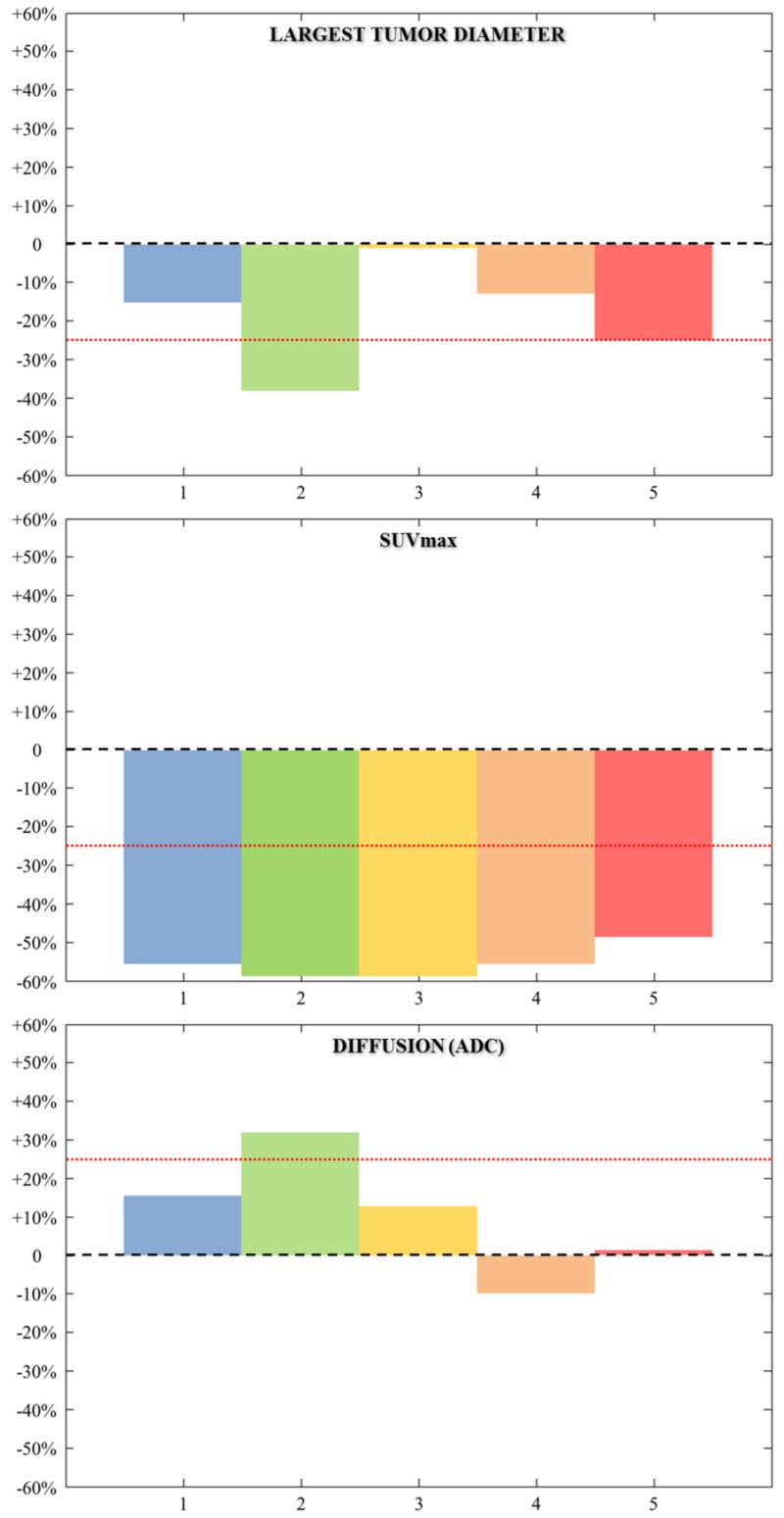
Graphical plots represent the variation (day 0 – day 8/day 0, in %) of the largest diameter, SUV^max^, and mean ADC within the lesion of the five patients studied (Each patient is represented by one color) Red dashed lines show 25% variation in ADC value (mean repeatability threshold derived from [30–33]. Patient 1 = blue box; patient 2 = green box; patient 3 = yellow box; patient 4 = brown box; patient 5 = red box.

Although exploratory, to investigate the possible clinical relevance of our pre-clinical findings, we took advantage of a previously reported window opportunity study that investigated the activity of cetuximab in previously untreated SCCHN patients. In this study, cetuximab was administered in monotherapy for two weeks prior to surgery. Five of the included patients had anatomical imaging and ^18^FDG-PET as well as DW-MRI evaluation at baseline and two weeks after cetuximab infusion. One patient achieved a partial response according to RECISTv1.1. Some degree of tumor shrinkage was found in three other patients (decrease in the largest tumor diameter by −16%, −13%, and −25%). All the patients had a ^18^FDG-PET partial response according to PET EORTC guidelines [17]. The only patient with an increase in ADC value above 25% was observed in the patient with the largest tumor shrinkage (Figure 5).

## DISCUSSION

We investigated the ability of 2′-deoxy-2′-[18F]fluoro-D-glucose-PET, DW-MRI and choline spectroscopy as a means to rapidly predict the activity of cetuximab, a mAb targeting the EGFR, in SCCHN PDTX models. To our knowledge, this is the first time that PDTX models have been used pre-clinically to investigate these imaging technologies. Our pre-clinical and clinical data suggest that DW-MRI and ^18^FDG-PET should be further investigated to predict cetuximab activity.

Preclinical and clinical studies support the use of ^18^FDG-PET to evaluate the activity of EGFR inhibition [15, 16, 23–28]. In some studies, a ^18^FDG-PET response was associated with tumor shrinkage or improved time to progression. Accordingly, in our study, cetuximab induced a significant decrease in SUVmax at day 8 compared to baseline in the most cetuximab-sensitive model (Cetux-S HNC004) but not in the other models. (Figure 2). At day 8, SUVmax was significantly lower in Cetux-S HNC004 and Cetux-S HNC002 compared to their respective controls (Figure 3). This could be explained by the respective tumor growth kinetics of the HNC004 and HNC002 derived models. Interestingly, at day 8, SUVmax was also significantly lower in Cetux-R HNC004 compared with controls despite the fact that the tumors were growing. This could be related to the speed of tumor growth that was still slower in Cetux-R HNC004 than that observed in the HNC004 control model treated with a saline solution. Furthermore, we have reported that cetuximab induced 90% of ^18^FDG-PET partial responses, according to the EORTC guidelines, in a window opportunity study where cetuximab was infused three times over a two-week period before surgery [29]. However, it is unlikely that 90% of the patients will achieve long-term benefit from cetuximab, as demonstrated in the large phase 3 trials that investigated this compound [9]. Therefore, further studies investigating later time points and/or more restrictive delta SUVmax cut-off are needed to try to identify the patients that will benefit from an anti-EGFR treatment.

Pre-clinical and clinical studies have shown that ADC increases after chemotherapy, radiotherapy, and targeted therapies [13, 18, 19]. Similarly, we recorded a significant increase in ADC in Cetux-S HNC004, the most sensitive cetuximab model. We did not find significant modifications in ADC values between baseline and day 8 in the resistant models (HNC010, Cetux-R HNC004, and Cetux-R HNC002, Figure 2) and in Cetux-S HNC002, the model where cetuximab has only moderate activity. Similar results were obtained when we compared the value between controls and cetuximab-treated groups at day 8 (Figure 3). These data suggest that DW-MRI could be an interesting imaging tool to predict sensitivity to anti-EGFR therapy even though it is yet to be investigated in the clinic. Although exploratory and limited by the very low number of patients, our clinical data support the hypothesis that ADC could be useful since we observed the highest increase in ADC (more than 25% compared with baseline) in the only patient with a partial response in our window study. The threshold of a 25% increase seems to be clinically relevant as suggested by previous works [30–33].

EGFR has been shown to activate the choline kinase-alpha or phosphatidylcholine-specific phospholipase C in some cancer models [34, 35]. Using optical imaging, pre-clinical data in breast cancer cell lines showed that gefitinib, an EGFR tyrosine kinase inhibitor, significantly reduced the uptake of choline metabolites in the sensitive cell line BT-474 but not in the resistant cell line MDA-MB-231 [36]. MEK inhibition has also shown to induce a significant drop in phosphocholine mediated by a decrease in the expression of choline kinase α [37]. Moreover, choline compounds involved in phospholipid synthesis and reflecting membrane turnover also show aberrant metabolism in cancer. Elevated tCho (total choline 1H MR signal at 3.2 ppm including contributions from choline, phosphocholine, and glycerophosphocholine) has been reported as a common feature in a large variety of cancers - the so called “cholinic phenotype” [38]. Changes in total choline (tCho) is therefore associated with positive responses in cancers in preclinical studies [21, 31, 39, 40]. We therefore postulated that EGFR inhibition could decrease the tCho pool. However, in our models, choline spectroscopy was not able to predict cetuximab activity. No significant decrease in the tCho pool was recorded at day 8 compared to baseline in the two sensitive models Cetux-S HNC004 and Cetux-S HNC002, even if a significant difference between Cetux-S HNC004 and controls was found at day 8. Several relationships exist between the choline cycle and cell-receptor activated signal transduction pathways, making the interpretation of the tCho spectral profile, in terms of pharmacodynamic biomarkers of targeted therapies, rather complex [35, 38]. Magnetic resonance spectroscopy (MRS)-detected effects of targeted agents in cancer cells do not provide any consensus on the type of change in the choline compounds observed, with both increases and decreases in tCho being associated with a positive response to different targeted therapies. Further pre-clinical work is needed to investigate this imaging technique as a predictor of response before it can be applied to clinical settings.

Some of the discrepancies observed in this work may be explained by the models used. Cetux-S HNC004 and HNC010 were primarily sensitive and resistant to cetuximab, respectively, and gave homogenous results. Cetux-S HNC002 was only moderately sensitive to cetuximab, with no tumor shrinkage compared to Cetux-S HNC004. Interestingly, within the Cetux-S HNC002 model, the recorded tumor growth was not homogenous with some mice experiencing faster tumor growth than others (data not shown). In addition, baseline pEGFR was low in HNC002, suggesting that this tumor might be less dependent on EGFR-related pathways for tumor growth. This could explain the large standard deviation observed and the absence of significant results in some of the HNC002 experiments. These observations underline the importance of using clinically relevant models to pre-clinically investigate the different imaging techniques before clinical investigation. Compared to high passage tumor cell lines, the models used in this study displaying heterogeneous responses might represent more clinically-realistic models. The high-passage tumor cell lines do not reflect the tumor heterogeneity observed in this work nor that encountered in patients.

There are some limitations in this work. First, the time-points used were based on previous literature suggesting that metabolic modifications could occur within one week after treatment [28]. We cannot exclude, however, that more predictable or homogenous results could be obtained at other time-points. Other FDG-PET parameters could have been used, although SUVmax is a familiar parameter that is frequently used in the clinic. Finally, the number of models employed in this study were low and do not represent the whole SCCHN cancer population. Nevertheless, we showed that these imaging techniques can be investigated in more relevant pre-clinical models than cancer cell lines. The intra-model and inter-model variations observed with our SCCHN PDTX outline the importance of tumor heterogeneity, and this should be taken into account when developing imaging technology to predict treatment response.

Altogether, our data support the ongoing investigation of metabolic imaging to predict treatment outcomes. It also supports the investigation of DW-MRI to predict the activity of anti-EGFR therapy. To our knowledge, and at the time of writing, this has never been undertaken.

## MATERIALS AND METHODS

### Generation of PDTX models

PDTX models were established in collaboration with Trace, the PDTX platform of KU Leuven (www.uzleuven-kuleuven.be/lki/trace), and derived from patients with SCCHN. Each patient signed an informed consent (ethics committee: UCL/MD/2012/09July/314). Mice were maintained and handled in accordance with the University catholique de Louvain policy for animal care. Patient tumor materials were collected in RPMI medium (Gibco, Waltham, MA, USA) supplemented with fungizone 0.4% (Bristol Myers Squibb, New-York, USA), Pen/Strep 2.5% (Sigma, St Louis, Missouri, USA), and gentamycin (Braun Medical, Bethlehem, Pennsylvania, USA), and kept at 4°C for engraftment within six hours of resection. Necrotic and supporting tissues were carefully removed using a surgical blade. Some tumor fragments were flash frozen and stored at −80° C for genomic profiling, and other fragments were fixed in 4% neutral-buffered formalin and paraffin embedded for histopathologic analysis. The remaining tumor fragments were implanted subcutaneously into the back of athymic nude female mice (NMRI-Foxn1nu, Taconic, NY, USA). Successfully engrafted tumor models were then passaged through several generations. Experiments were conducted on the fifth and sixth generations. Models were validated by comparing the clinical behavior (sensitivity to cetuximab), gene expression profile (RNAsequencing) and immunochemistry (p16 (clone G175-405, BD Pharmingen, CA, USA); Ki67 (polyclonal rabbit, Thermo Fisher Scientific, Waltham, MA USA); p53 (clone SP5, Thermo Fisher Scientific, Waltham, MA USA); pEGFR (clone 7A5, Cell Signaling Technology, MA, USA); vimentin (clone SP20, Thermo Fisher Scientific, Waltham, MA USA), and E-Cadherin (clone 24E10, Cell Signaling Technology, MA, USA)) of the primary tumor with the tumors harvested from fourth and sixth generation mice. Only models with good concordance were used.

Some mice from the initial HNC002 and HNC004 models were treated with cetuximab (30 mg/kg, once a week intraperitoneally) until some became resistant to cetuximab. Resistance was defined as continuous tumor growth under cetuximab and an increase in tumor volume of more than 200% compared with baseline. The resistant models were treated continuously with cetuximab without interruption.

Animal work was undertaken in compliance with the Belgian law and all the experiment were realized in accordance with our local ethical committee. Animal welfare is regularly controlled by inspections in adherence with the Belgian law and all investigators performing animal work successfully completed FELASA C training.

### Treatment

Cetuximab sensitive (Cetux-S) and resistant (Cetux-R) SCCHN bearing mice (tumor size: +/-200 mm³) were treated intraperitoneally with cetuximab (Merck Serono, Darmstadt, Germany) at a dose of 30 mg/kg. Two doses of cetuximab were given: the first was administered just after the initial ^18^FDG-PET and MR assessments at baseline (day 0), and the second followed one week later on day 7. Post-treatment MR and ^18^FDG-PET were performed on days 1 and 8. The vehicle (saline solution) was administrated in the same conditions to the control groups (CTL). Mice were distributed randomly in the different groups. For PET and MRI experiments, the investigators were blinded as to treatment group both at acquisition and at analysis. Tumor size was measured by caliper once a week and tumor volume was calculated according to the following equation:

$$\text{V}\left({\text{mm}}^{3}\right)=\frac{\left(\text{the largest length}\right)\text{×}{\left(\text{the shortest length}\right)}^{2}}{2}$$

### 2′-deoxy-2′-[18F]fluoro-D-glucose-PET experiments

Small-animal PET scanner (Mosaic, Philips Medical Systems, Cleveland, USA) with a spatial resolution of 2.5 mm (FWHM) was used to perform PET imaging. Fasting animals were anesthetized by isoflurane inhalation (2.5% in air for induction and 1–2% in air for maintenance) and body temperature was maintained with a flow of warm air throughout the anesthesia period. Anesthetized mice were injected intraperitoneally with 100 μl (200 to 300µCi) of 2′-deoxy-2′-[18F]fluoro-D-glucose (10 mCi/mL; Betaplus Pharma, Brussels, Belgium) diluted in saline. For attenuation correction, a 10-minute transmission scan was performed in single mode using a 370 MBq ^137^Cs source, followed by a 10-minute static PET acquisition started 60 minutes after FDG injection. After correction of raw data for attenuation, random and scatter coincidences and for system dead-time, images were reconstructed using a fully 3D iterative algorithm (3D-RAMLA) with a voxel size of 1 mm^3^. After PET acquisition, anesthetized mice were transferred on the same bed to the computed tomography (CT) scanner (NanoSPECT/CT small Animal Imager, Bioscan, USA) for anatomical reference. Regions of interest were manually delineated on PET images using PMOD software (PMOD^™^, version 3.5, PMOD technologies Ltd, Zurich, Switzerland) and FDG uptake was expressed as SUVmax defined as the maximal uptake in tumor normalized to injected dose par unit weight of mice.

### MR experiments

MR experiments were performed in an 11.7-Tesla, 16-cm inner diameter bore system (Bruker, Biospec, Ettlingen, Germany) equipped with a quadrature volume coil (40-mm inner diameter). Mice were anesthetized by isoflurane inhalation under the same conditions as during the PET experiments. Body temperature was maintained using a warm circulating water blanket and was checked using a rectal temperature probe. A pressure cushion was used to monitor breathing, allowing adaptation of anesthetic gas flow when needed.

As well as providing reference images, anatomical T2-weighted images were used to assess tumor volume and largest tumor diameter for further single voxel spectroscopic acquisitions. This turbo RARE sequence had the following parameters: repetition time (TR) = = 2.417s, echo time (TE) = 33 ms, averages = 1, field of view = 4 × 4 cm, 18 slices with a 1 mm thickness. Optimization of magnetic field homogeneity (localized shimming) was performed until a linewidth of water resonance below 50 Hz was achieved. Manual water suppression (VAPOR) was used. ^1^H-MR spectra were acquired using a point-resolved spectroscopy (PRESS) localization technique, with the following parameters: TR = 2.5 s, TE = 20 ms, signal averages = 256, voxel size = 3 × 3 × 3 mm^3^, and total acquisition time = 10 min 50 s. Spectra obtained using this technique were analyzed using jMRUI software version 5.0. Metabolite model signals used in quantitation based on quantum estimation (QUEST) were simulated in NMR-SCOPE (NMR spectra calculation using operators; jMRUI). Signals were imported in jMRUI, pretreated by Hankel Lanczos Singular Value Decomposition (HLSVD) to eliminate any residual water peak, and rephased. Model fitting was performed using the QUEST routine of jMRUI. Peak areas were measured for tCho peak (δ = 3.2 ppm) and normalized with the water peak area (δ = 4.7 ppm) from a non-water suppressed scan using a same volume of interest and geometry.

For Diffusion Weighted-MRI, a transverse echo planar imaging sequence was used with the following parameters: TR/TE = 3000/27 ms, duration of diffusion gradients δ = 7 ms, separation of diffusion gradients (Δ) = 14ms, slice number = 7, slice distance = 1 mm, *b*-values = 0–100–200–400–600–800–1000 s/mm^2^, acquisition time = 4 min 12 sec. Mean apparent diffusion coefficients (ADC) were extracted from DW images and averaged for every slice of tumor using Matlab software (The MathWorks Inc., Natick, MA, USA).

The exponential decay of the signal as a function of the b-value was measured according to the Stejskal–Tanner equation. ADC maps were generated by nonlinear least squares regression of a mono-exponential to the experimental signal intensity for all *b* values.

### pEGFR Immunohistochemistry (IHC)

pEGFR IHC (clone 7A5, Cell Signaling Technology, MA, USA) was performed on 4-µm paraffin embedded tumor sections. Slides were scanned (Leica SCN400 Slide Scanner, Meyer, USA) and analysed using Slide Path program. Expression was subsequently quantified at 40 times magnification by measuring the staining intensity and the number of positive tumor cells expressed as a percentage. A histoscore with a potential range of 0–300 was calculated as follows: Histoscore = (% weakly stained cells) + (% moderately stained cells) × 2 + (% strongly stained cells) × 3 [41].

### Patients

Cetuximab was administered for two weeks prior to surgery to 33 treatment-naïve patients (NCT00714649). Details of the eligibility criteria, pretreatment evaluation, safety, and clinical results have been published [29]. The clinical and translational parts of the study were approved by the Independent Ethics Committee and the Belgian Health Authorities, and conducted in accordance with the Declaration of Helsinki (October 2000). Five patients in this study had DW-MRI, ^18^FDG-PET, and anatomical tumor evaluation by RECISTv1.1. The imaging guidelines employed have been previously described [29], (Supplementary Data 1).

### Statistics

The two primary endpoints of this study aimed to determine (i) if the imaging parameters experienced significant changes between baseline and day 8 and (ii) if these changes differed between the cetuximab treated groups versus the untreated group in each model at day 8.

All analyses were performed using GraphPad Prism 7 software. The parameters of largest tumor diameter, SUVmax, total choline to water ratio and ADC were found to be normally distributed according to Shapiro Wilk normality test. Mean data at baseline and day 8 were then compared using an independent samples *t*-test. Comparisons of changes in imaging parameters (normalized to their baseline values) between control, sensitive, and resistant groups in each model were carried out using the one-way ANOVA test followed by Tukey test for pairwise comparisons. Two-way ANOVA analysis (fixed effects: time and group) and multiple comparison post-tests (Tukey) were performed to compare tumor growth in control, sensitive, and resistant groups.

### All tests cited above were 2-sided, and a *p*-value < 0.05 was regarded as statistically significant

The number of mice to include per group were calculated using the following hypotheses: *N* = 2 × σ^2^/Δ^2^ × f (α, β) (N: number of mice per group; σ: Standard deviation of data; Δ: size of difference, minimal effect of interest; α: 0.05, β: 0.8). Therefore, the minimum number of mice per group was 6.

## SUPPLEMENTARY MATERIALS FIGURES



## Abbreviations

18FDG-PET: 2′-deoxy-2′-[18F] fluoro-D-glucose positron emission tomography
DWI-MRI: diffusion-weighted magnetic resonance imaging
SCCHN: squamous cell carcinoma of the head and neck
