# Supplementary material for: 2′-deoxy-2′-[18F] fluoro-D-glucose positron emission tomography, diffusion-weighted magnetic resonance imaging, and choline spectroscopy to predict the activity of cetuximab in tumor xenografts derived from patients with squamous cell carcinoma of the head and neck
Source: Oncotarget. 2018 Jun 19;9(47):28572–85. doi: 10.18632/oncotarget.25574 (PMC6033354; doi:10.18632/oncotarget.25574)
Supplement: Supplementary file 1 [file oncotarget-09-28572-s001.pdf]

2′-deoxy-2′-[18F] fluoro-D-glucose positron emission tomography, diffusion-weighted magnetic resonance imaging, and choline spectroscopy to predict the activity of cetuximab in tumor xenografts derived from patients with squamous cell carcinoma of the head and neck

SUPPLEMENTARY MATERIALS

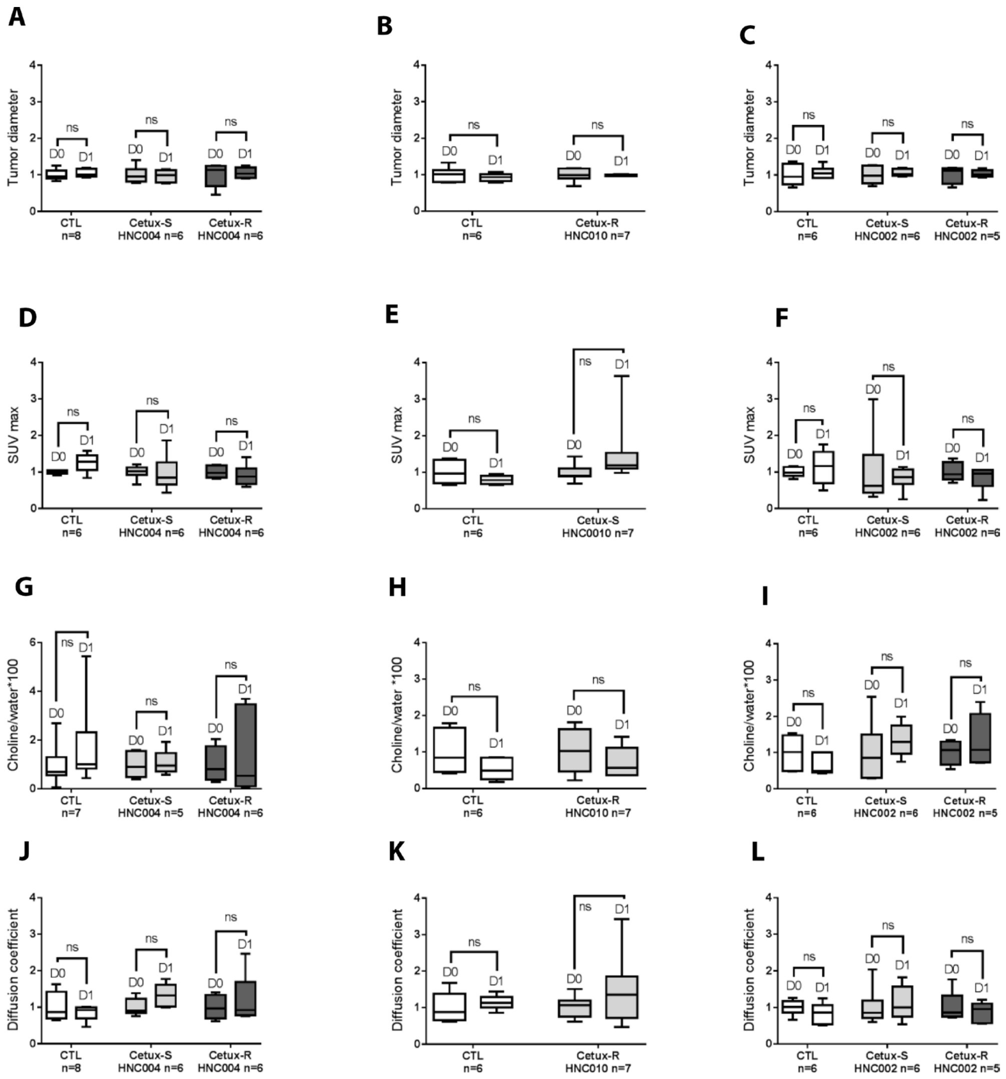

Supplementary Figure 1: Change in imaging parameters between baseline and day 1 in each model (Box Plot). (**A–C**) Modifications of the largest tumor diameter between baseline and day 1 in each group. (**D–F**) Evolution of SUV max (standard uptake value) at day 1 compared to baseline in the different models. (**G–I**) Changes in Choline/water*100 (total choline to water ratio inside the tumor) between day 0 and day 1 in each model. (**J–L**) Evolution of apparent diffusion coefficient inside the tumor at day 1 compared to baseline. CTL = control mice of each model treated with saline solution; Cetux-S HNC004 = Cetux-S HNC004 mice treated with cetuximab; Cetux-R HNC004 = Cetux-R HNC004 mice treated with cetuximab; HNC010 = HNC010 mice treated with cetuximab; Cetux-S HNC002 = Cetux-S HNC002 mice treated with cetuximab; Cetux-R HNC002 = Cetux-R HNC002 mice treated with cetuximab. Thirty mg/kg of cetuximab was given intraperitoneally on day 0 and 7.

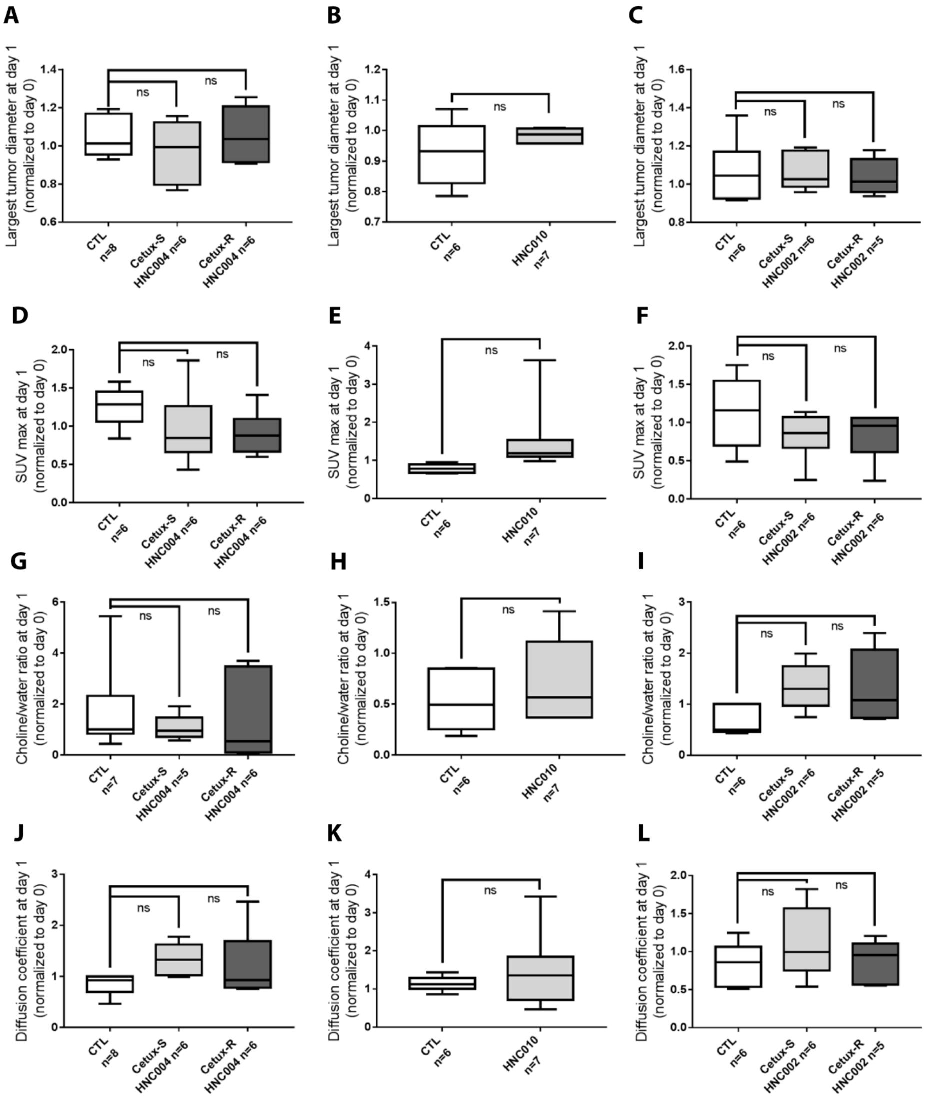

Supplementary Figure 2: Comparison of the imaging parameters between different groups in each model at day 1 (Box Plot). (**A–C**) In each model, comparison of the largest tumor diameter at day 1 between different groups and their respective control. (**D–F**) In each model, comparison of the SUV max (standard uptake value) at day 1 between different groups. (**G–I**) At day 1, comparison of the choline/water ratio between different groups. (**J–L**) Comparison of the apparent diffusion coefficient at day 1 between different groups and their respective control. CTL = control mice of each model treated with saline solution; Cetux-S HNC004 = Cetux-S HNC004 mice treated with cetuximab; Cetux-R HNC004 = Cetux-R HNC004 mice treated with cetuximab; HNC010 = HNC010 mice treated with cetuximab; Cetux-S HNC002 = Cetux-S HNC002 mice treated with cetuximab; Cetux-R HNC002 = Cetux-R HNC002 mice treated with cetuximab. Thirty mg/kg of cetuximab was given intraperitoneally on day 0 and 7.
